# M5542, a bifunctional fusion protein targeting CD80, CD86, and OX40L that modulates excessive T-cell activity: a preclinical proof-of-concept study

**DOI:** 10.3389/fimmu.2026.1809074

**Published:** 2026-08-26

**Authors:** Michelle Downing, Allison Christiaansen, Ling Zhang, Katherine Roe, Namita Kumar, Aditee Deshpande, Hong Zhang, Ohad Tarcic, Mira Toister-Achituv, Maria Soloviev, Alec W. Gross, Gang Chen, Chia Chi Sun

**Affiliations:** 1 Research Unit – Neuroscience and Immunology, EMD Serono Research and Development Institute, Inc., an affiliate of Merck KGaA, Billerica, MA, United States; 2 Discovery Development Technologies, Inter-Lab Ltd., an affiliate of Merck KGaA, Yavne, Israel; 3 Discovery Technologies, EMD Serono Research and Development Institute, Inc., an affiliate of Merck KGaA, Billerica, MA, United States

**Keywords:** anti-OX40L, autoimmunity, co-stimulatory pathways, CTLA-4Ig, inflammation, pre-clinical study

## Abstract

**Background:**

Chronically activated T-effector (Teff) cells can play a pivotal role in T-cell-mediated diseases including systemic lupus erythematosus subsets, lupus nephritis and graft-vs-host disease (GvHD). Since T cells require diverse costimulation signals to fully activate, proliferate, and differentiate into Teffs, we hypothesized that modulating two costimulatory pathways (CD28 and OX40) with a single bifunctional molecule would provide better control of pathogenic Teffs in autoimmune diseases than currently available T-cell costimulation modulators. We assessed the effects of dual-pathway blockade by M5542, a novel CD80, CD86, and OX40L antagonist and bifunctional fusion molecule. We determined the advantages of M5542 blocking both CD28 and OX40 pathways over single-pathway blockade in *in vitro* potency and *in vivo* studies, to more effectively curb T-cell-mediated inflammation in autoimmune diseases.

**Methods:**

M5542, single-agent comparators CTLA-4Ig (abatacept), anti-OX40L, and both single agents in combination were tested for their ability to inhibit proinflammatory cytokine production in a mixed lymphocyte reaction of activated monocyte-derived dendritic cells and T cells from healthy donors or peripheral blood mononuclear cells from individuals with SLE. The target occupancy of CD80 and OX40L on dendritic cells was assessed by flow cytometry. The effects of M5542 and comparators on T-cell-mediated inflammation were assessed using a keyhole limpet hemocyanin (KLH) peptide-induced delayed-type hypersensitivity (DTH) humanized hOX40/hOX40L mouse model. All molecules were also tested for their efficacy to reduce disease in a humanized xenogenic GvHD (xGvHD) mouse model.

**Results:**

M5542 potently reduced proinflammatory cytokine production and dampened T-cell proliferation compared with CTLA-4Ig or anti-OX40L *in vitro*. M5542 also neutralized OX40L-mediated T-regulatory cell (Treg) dysfunction *in vitro* and suppressed Teff proliferation in conjunction with Tregs *in vitro*. Moreover, M5542 dose-dependently reduced T-cell-mediated ear swelling and anti-KLH antibody production in the KLH-DTH mouse model. In the xGvHD model, M5542 demonstrated improved efficacy over CTLA-4Ig or anti-OX40L alone in suppressing human IFNγ and preventing disease.

**Conclusion:**

In this preclinical study, the bifunctional M5542 blocked CD28 and OX40-mediated T-cell inflammation with greater immunomodulatory activity than monofunctional agents and in some assays comparable to or better than the combination of the monofunctional agents. These findings support further evaluation of M5542 in T-cell-driven autoimmune disease settings.

## 1 Introduction

Uncontrolled and chronically activated T cells underlie many autoimmune diseases (1). Their unchecked activity can lead to organ damage and patient mortality (2, 3). T cells depend on a variety of costimulation signals to fully activate, proliferate, and differentiate into T-effector (Teff) cells (4); therefore, modulating costimulation pathways offers an effective treatment for T-cell-driven autoimmune diseases.

T cells become fully activated when they receive two signals: 1) T-cell receptor activation due to interaction with a peptide-major histocompatibility complex on the surface of an antigen-presenting cell (APC), and 2) T-cell costimulatory receptor activation due to interaction with their cognate ligands (5, 6). T-cell stimulation in the absence of the second costimulatory signal results in the development of anergic or tolerogenic T-cell responses (5). There are two major groups of costimulatory molecules, the B7-CD28 (7) and the tumor necrosis factor receptor (TNFR) superfamilies (8), and their variety of expression patterns, whether constitutive or inducible, all contribute to enhance the heterogeneity of and fine-tune the response of the cellular immune repertoire and are extensively reviewed here (7–9). Depending on the autoimmune disease, stage of disease, or autoantigens, the temporal induction of costimulation molecules expressed on APCs or costimulatory receptors on T cells can be exploited for selective immunomodulation (9). Due to the heterogeneity of autoimmune diseases where multiple autoantigens are presented to different T-cell subsets utilizing different costimulatory ligand–receptor pairs, we hypothesize that targeting two ligand-receptor pathways blocks these heterogenous T-cell responses more effectively than targeting one.

CD80/CD86 (B7-1/B7-2) are well-studied costimulatory ligands which are upregulated on APCs such as B cells, monocytes, macrophages, and dendritic cells during inflammatory conditions. These ligands engage CD28 on T cells to provide a second costimulatory signal to activate naïve T cells and promote proliferation. Abatacept, a recombinant fusion protein comprising human immunoglobulin G (IgG) and the extracellular domain of cytotoxic T lymphocyte-associated protein 4 (CTLA-4Ig), selectively binds to CD80/CD86 on APCs effectively preventing CD28 signaling on T cells, attenuating T-cell activation and proliferation (10–12). Abatacept is clinically approved for the treatment of autoimmune diseases including adult rheumatoid arthritis (RA), psoriatic arthritis, and juvenile idiopathic arthritis as well as prophylaxis of acute graft vs host disease (GvHD) (13, 14). However, in clinical trials for systemic lupus erythematosus (SLE), lupus nephritis (LN), and primary Sjogren’s Syndrome, abatacept failed to meet its clinical endpoints. While it demonstrated efficacy in dampening newly activated T cells and follicular helper T cells (15–17), CTLA-4Ig was less effective in targeting memory T cells or terminally differentiated CD28 null T cells, which depend on other costimulatory pathways, including the OX40-OX40L signaling pathway (18).

The therapeutic potential of targeting the OX40-OX40L signaling pathway has been demonstrated in both preclinical disease models and clinical trials. In response to inflammatory signals, the OX40 ligand (OX40L, also known as CD252, gp34, and TNFSF4) is induced on the surface of APCs such as dendritic cells (9, 19, 20). The OX40 receptor (CD134, TNFRSF4) is expressed on activated CD4 and CD8 T cells or activated memory T cells, but not on resting naïve or resting memory T cells (9, 21). Binding of OX40L to OX40 stimulates T-cell expansion, differentiation, and survival of Teff subsets including T helper 1 (Th1), T helper 2 (Th2), T follicular helper (Tfh), and T helper 17 (Th17) cells (9, 21, 22). OX40 signaling is also critical for the generation, maintenance, and optimal reactivation of memory CD4+ T-cells (23). Forkhead box P3 (FOXP3)+ T-regulatory cells (Tregs) play a critical role in suppressing overactivated effector T cells. However, in autoimmune diseases OX40L-OX40 signaling contributes to dysregulation of Treg function, further amplifying the imbalance of pathologic T effectors. Moreover, OX40 signaling is known to suppress the generation of induced Tregs (iTregs) by decreasing FOXP3 expression (9, 24, 25). Dysregulation of the OX40L-OX40 pathway has been implicated in the pathogenesis of SLE and LN, Th2-driven allergic responses, and atopic dermatitis (26–30).

Amlitelimab (SAR-445229, KY-1005) is an anti-OX40L monoclonal IgG4 antibody which has demonstrated efficacy in Phase 2 studies in atopic dermatitis (31, 32), showcasing the benefit of interrupting OX40L-driven Th2 inflammation. However, targeting the OX40L-OX40 pathway alone fails to address the continued generation and maintenance of activated Th2 cells and other T-cell subsets through the CD80/CD86-CD28 pathway. Dual inhibition of both signaling pathways could offer potential improvements in efficacy.

We have designed M5542 (CTLA-4Ig_anti-OX40L) as a non-depleting and non-cytotoxic bifunctional T-cell immunomodulator. It is a CD80, CD86, and OX40L antagonist fusion molecule comprising CTLA-4Ig fused to two anti-OX40L antigen binding fragments (Fab) (**Supplementary Figure 1**; see Online Resource), to provide dual blockade of the CD80/86-CD28 and OX40L-OX40 signaling pathways. We hypothesize that M5542, as a dual-pathway blocker, would more efficiently inhibit T-cell activation, differentiation, and proliferation than either single-pathway blockade with CTLA-4Ig or anti-OX40L alone. In the present study, we evaluated M5542 across *in vitro* and *in vivo* preclinical models, demonstrating its potential advantage compared with monofunctional agents in reducing T-cell-mediated inflammation and supporting its evaluation in autoimmune disease settings.

## 2 Materials and methods

### 2.1 Materials

The structure and sequence information for M5542, described in an international PCT patent publication WO 2022/261079 A2, consists of the extracellular domain of CTLA-4 fused to human IgG1 containing effector silencing mutations (that abrogate antibody-dependent cytotoxicity, antibody-dependent cellular phagocytosis, and complement-dependent cytotoxicity) and anti-OX40L Fabs. A schematic representation of the M5542 molecule is provided in **Supplementary Figure 1**.

The anti-OX40L monoclonal antibody is based on clone 02D10 as disclosed in US9139653B1 generated in human IgG1 format to facilitate comparability with M5542. All experiments presented in the manuscript were performed using this clone (herein referred to as anti-OX40L).

CTLA-4Ig (abatacept, Bristol–Myers Squibb), a fusion protein consisting of the extracellular domain of CTLA-4 and modified Fc portion of human IgG1 that eliminates complement activation, was purchased commercially and used as a comparator in all experiments presented in the manuscript. Isotype control, anti-hen egg lysozyme (HEL) IgG1 containing the same Fc silencing mutations as M5542, was used as a comparator in all experiments presented.

### 2.2 Human CD80, CD86, and OX40L steady-state affinity assay using surface plasmon resonance

Surface plasmon resonance (SPR) experiments to determine the binding of M5542 to recombinant human CD80 (Novoprotein cat.# CK61) and CD86 (Novoprotein cat.# C404) were performed using a Biacore T200 or Biacore 8K+ system (GE Healthcare) and on a Biacore T200 to determine M5542 binding to OX40L. All binding assays were performed on the CM5 series S sensor chip.

M5542, CTLA-4Ig, and an isotype control were immobilized on the sensor chip via goat anti-human IgG Fc (50 µg/mL at pH 5.0, Jackson ImmunoResearch, cat.# 109-005-098). Ligand capture for 1 µg/mL was in the 200–400 resonance unit (RU) range. Human CD80 and CD86 were bound to this surface over a range of 10 concentrations (0 to 2,000 nM) using serial twofold dilutions from 2,000 nM. Association was measured for 900 s, and dissociation was measured for 300 s, both at a flow rate of 10 µL/min at 10 °C.

To assess binding of M5542 to OX40L, recombinant OX40L proteins were immobilized onto the sensor chips at 250 RU. The analyte proteins M5542, anti-OX40L, and isotype control IgG were bound to this surface at a range of 0 to 125 nM using serial twofold dilutions from 125 nM. Association was measured for 900 s and dissociation for 180 s using a flow rate of 10 µL/min at 10 °C.

The response at steady state was plotted against the concentration of recombinant protein or antibody and fitted to a 1:1 binding model. The steady state, K_D_, was determined as the recombinant protein (for CD80 and CD86) or antibody (OX40L) concentration at a response equal to half the maximal response (R_max_).

### 2.3 Human biological samples

Human leukocytes isolated from peripheral blood were used for various *in vitro* assays. Buffy coats from anonymized healthy volunteers were collected from the New York Blood Center, New York, NY for research purposes.

Heparinized whole blood samples from individuals with SLE were collected by Sanguine Biosciences. Institutional Review Board approval for the collection of anonymized samples was provided via Advarra (IRB00000971). All individuals provided informed consent and were confirmed diagnosed with SLE per deidentified medical records. Isolated peripheral blood mononuclear cells (PBMC) from three individuals with moderate-severe symptoms on standard of care (hydroxychloroquine) and self-reported ongoing flare or had a flare within the last 6 months were tested in the SLE PBMC mixed lymphocyte reaction (MLR) assays.

### 2.4 MLR assays

#### 2.4.1 PBMC isolation

All blood samples were used within 18 h of collection. To isolate PBMCs, whole blood or buffy coats (diluted twofold in phosphate-buffered saline [PBS]) were separated via density gradient centrifugation (720 g for 20 mi) using Ficoll-Paque (GE Healthcare). Isolated PBMCs were washed twice with PBS and resuspended in culture media for use in experiments.

#### 2.4.2 Monocyte-derived dendritic cell generation and activation

Monocytes were isolated from healthy volunteer PBMCs using the Human Pan Monocyte Isolation Kit (Miltenyi Biotec) according to the manufacturer’s instructions. Isolated monocytes were washed in PBS, resuspended in 10 mL of AIM-V media with 5% human AB Serum and PenStrep (Gibco, Human AB Serum [Sigma], Penicillin Streptomycin [PenStrep; Invitrogen]), and counted using trypan blue. Monocytes were then resuspended at 10^6^ cells/mL in AIM-V media with 5% human AB Serum and PenStrep with 50 ng/mL granulocyte-macrophage colony stimulating factor (GM-CSF) and 50 ng/mL interleukin (IL)-4 for 5 days at 37 °C in 5% CO_2_. Monocyte-derived dendritic cells (MDDCs) were activated according to Arimoto-Miyamoto et al. (33), with 2 µg/mL PGE2 (Sigma), 40 ng/mL TNFα (PeproTech), 40 ng/mL IL-6 (R&D Systems), and 20 ng/mL IL-1β (BD Biosciences) and incubated for 2 days at 37 °C, 5% CO_2_. Expression of CD80, CD86, and OX40L (BioLegend, 305222, BD Biosciences 561128, 558164) was measured via flow cytometry on an LSR Fortessa X20 (BD Biosciences) to confirm the activation status of MDDC.

#### 2.4.3 MDDC: T-cell coculture

Total T cells were isolated from freshly isolated healthy volunteer PBMCs by negative selection using the Human Pan T-cell Isolation Kit (Miltenyi Biotec) according to the manufacturer’s instructions. T cells were stained with CellTrace Violet (CTV) (Thermo Fisher) and resuspended at 1 × 10^6^ cells/mL in complete RPMI (Gibco, 10% fetal bovine serum (Corning), PenStrep (Invitrogen)). MDDCs (stimulators) and T cells (responders) were cultured at a 1:4 ratio in a 96-well flat-bottom plate (Falcon) with fourfold serial diluted test agents M5542, CTLA-4Ig, anti-OX40L, or the combination 1:1 of anti-OX40L+CTLA-4Ig from a starting concentration of 1000 nM and incubated for 4 days at 37 °C. Supernatant from six experiments was collected on day 2 for IL-2 assessment (AlphaLISA AL221C, Perkin Elmer) and day 4 for TNFα, interferon (IFN)γ and IL-6 assessment (AlphaLISA AL208C, AL217C, AL223C, Perkin Elmer).

To determine receptor occupancy, MDDCs from two MLRs were harvested on day 4 and stained with the following antibodies against CD11c (BioLegend, 301604), CD4 (BioLegend, 357406), CD8 (BD Biosciences, 563795), CD80 (BioLegend, 305222), and OX40L (BD Biosciences 558164) and measured by flow cytometry using an LSR Fortessa X20 (BD Biosciences). MDDCs (defined as CD14^-^CD11c^+^ cells), were analyzed for free % CD80 or OX40L positive cells using FlowJo v10.8. Receptor occupancy was calculated by the equation 100% − free % CD80 or OX40L detected on the MDDCs in each test condition and plotted using GraphPad Prism 10. The MDDC: T-cell isotype control condition was used as 0% CD80/OX40L receptor occupancy or maximum detection of free % CD80/OX40L. 100% CD80 or OX40L occupancy corresponds to complete blocking of the target by test agents unlabeled CTLA-4Ig/anti-OX40L at the highest concentration (where free% CD80/OX40L expression is 0%).

For proliferation readouts, cocultures were set up as above, but instead of T cells as responders, PBMCs were used and stained with CTV (Thermo Fisher, C34557) and anti-CD3 beads (StemCell Technologies, 10309) were added. At day 4, cells from three experiments were harvested and stained for CD4 (BioLegend, 357406), CD8 (BD Biosciences, 563795), CD28 (BioLegend, 302966), OX40 (BD Biosciences, 563664) and CD11c (BioLegend, 301604) and measured by flow cytometry using LSR Fortessa X20 (BD). CD4 and CD8 proliferation was assessed by CTV dilution and analyzed by FlowJo v10.8. The percentage of CD4 and CD8 proliferating cells and the percentage of OX40+CD28+ cells within the CD4+ or CD8+ cell populations were plotted using GraphPad Prism. 10.

#### 2.4.4 MDDC: SLE PBMC coculture

PBMCs isolated from three individuals with SLE were resuspended at 1 × 10^6^ cells/mL in complete RPMI and cultured with healthy activated MDDCs at a ratio of 4:1. Healthy donor–derived MDDCs were used to ensure sufficient antigen-presenting cells and were activated with a cytokine cocktail to model inflammatory conditions and to induce the expression of CD80/CD86 and OX40L as described by Arimoto-Miyamoto et al. (33) Test agents M5542, CTLA-4Ig, anti-OX40L, or the combination 1:1 of anti-OX40L+CTLA-4Ig were added at a final concentration of 50 or 1 nM and incubated at 37 °C in 5% CO_2_ for 4 days. Supernatant from three experiments were collected for cytokine analysis on day 4 for measurement of TNFα and IFNγ by AlphaLISA, or for analysis of IL-4, IL-13, Granzyme B, and GM-CSF using a custom human Proinflammatory Panel (MSD).

### 2.5 Internalization assay

Internalization of M5542, CTLA-4Ig, and anti-OX40L by MDDCs was measured in real time using live-cell imaging. Activated MDDCs were incubated with 100 nM pHrodo-labeled M5542, CTLA-4Ig, or anti-OX40L, with pHrodo-labeled isotype control and the anti-CD20 (rituximab) as negative controls. When internalized, the pHrodo dye fluoresces at low pH, allowing internalization to be monitored. Real-time internalization was measured over 20 h using the Celldiscoverer 7 live-cell imaging system (Zeiss). Two separate experiments were performed on one donor MDDC.

### 2.6 iTreg induction assay

Fresh PBMCs were isolated from five buffy coats using a Ficoll gradient as described above, and then naïve CD4+ T cells were isolated from PBMCs using the naïve CD4 Human T-cell isolation kit (Miltenyi) according to the manufacturer’s instructions. Naïve CD4+ T cells were cultured *in vitro* in complete RPMI 1640 medium (supplemented with 10% heat-inactivated fetal bovine serum, 2 mM L-glutamine, 50 IU penicillin/50 µg/mL streptomycin, 1 mM Na pyruvate, 55 µM β-mercaptoethanol, and 0.01 M HEPES), plated at a concentration of 100,000 cells per well in a 96-well tissue culture plate and stimulated with plate-bound anti-CD3 (5 µg/mL) and soluble anti-CD28 (1 µg/mL), plus TGF-β (5 ng/mL) and IL-2 (50 U/mL). The cells were incubated for 5 days at 37°C in 5% CO_2_. Soluble OX40L (1 µg/mL) was added to most conditions except controls. Either isotype control IgG or test agents M5542, CTLA-4Ig, or anti-OX40L (50 nM) were added to respective wells on day 0. Cells from five experiments were collected and intracellular FOXP3 staining was performed using eBioscience instructions for “Staining Intracellular FOXP3”. These samples were analyzed using a BD LSR Fortessa X-20. Data analysis was performed using FlowJo software. Tregs were identified based on phenotype of CD25^hi^ CD127^low^ FOXP3^+^.

### 2.7 Three-way MDDC: Treg : Teff coculture

Treg suppression assays were performed using Tregs and Teff cells isolated from two healthy donor PBMCs. Allogeneic MDDCs were used to activate each paired Treg/Teff cell in two experiments. CD4^+^ T cells were isolated from freshly prepared PBMCs using a human CD4 T-cell isolation kit (Miltenyi) according to the manufacturer’s instructions and sorted into CD4^+^, CD25^hi^, CD127^low^ Treg, and CD4^+^ CD25+CD127+ non-Tregs (Teff) using the FACSAria cell sorter. Tregs were labeled with 2 µM of CTV, and CD4^+^ Teff cells were labeled with 2 µM CellTrace CFSE dye. MDDC (2.5 × 10^4^ cells per well) and Teff cells (5 × 10^4^ cells per well) were plated in 96-well tissue culture plates and incubated in complete AIM-V medium supplemented with 5% human AB serum, soluble anti-CD3 (0.6 µg/mL), and PenStrep. Treg cells (1.25 × 10^4^ per well) were added together with 10 nM of M5542, CTLA-4Ig, anti-OX40L, isotype control, or media control, cultured for 5 days at 37 °C in 5% CO_2_ for 4–5 days and then analyzed by flow cytometry using the LSRFortessa X-20 (BD Biosciences). The immunostaining panel consisted of CD4 (BD Biosciences, 560837) and CD11c (BD Biosciences, 565227). Teff proliferation was assessed by CFSE dilution, and the histograms were analyzed using FlowJo.

### 2.8 Mice

Female, 6–8-week-old C57BL/6-Tnfrsf4tm1^(TNFRSF4)Bcgen^Tnfsf4tm1^(TNFSF4)Bcgen^/Bcgen (hOX40/hOX40L) mice were purchased from Biocytogen for the keyhole limpet hemocyanin (KLH) model. Female, 7–8-week-old NSG mice (NOD-*scid* IL2Rgamma^null^) were purchased from Jackson Labs (Bar Harbor, ME) for the xenogenic GvHD (xGvHD) model. All procedures related to animal handling, care, and treatment during the study were performed according to the guidelines approved by the institutional animal care and use committees of EMD Serono and following the guidance of the Association for Assessment and Accreditation of Laboratory Animal Care.

### 2.9 KLH-delayed type hypersensitivity (KLH-DTH) humanized hOX40/hOX40L mouse model

Humanized OX40/OX40L female mice (C57BL/6-Tnfrsf4tm1^(TNFRSF4)Bcgen^Tnfsf4tm1^(TNFSF4)Bcgen^/Bcgen (hOX40/hOX40L), Biocytogen) aged 6–8 weeks were treated with M5542 1, 3, or 10 mg/kg or isotype control 10 mg/kg 1 day prior to immunization and three times per week subsequently; eight mice per group. Cyclosporin A was administered orally at 60 mg/kg from days −1 to 15; eight mice per group. As negative control for immunization, Complete Freund’s Adjuvant (CFA) only was administered; four mice per group. Mice were immunized with 100 µL of 0.5 mg/mL KLH peptide (Millipore Sigma H7017) in a 50% PBS-CFA (InvivoGen) emulsion, and 7 days post-immunization received 10 µL of 1 mg/mL KLH peptide (Millipore Sigma H7017) via injection into their left ear. Ear thickness was measured under anesthesia at baseline and daily after the injection of KLH peptide. Animals were euthanized at 15 days post-immunization. Plasma was collected for anti-KLH Ab detection. Ear tissue samples were collected for histology.

#### 2.9.1 Immunohistochemistry and digital image analysis

Five-micron-thick tissue sections were generated from formalin-fixed paraffin embedded ear tissue samples, and CD4 (ab183685) immunohistochemistry was conducted via the automation platform Leica Bond-RX™ with the ready-to-use Bond Polymer Refine detection (DS9800) system. The system is biotin free and contains a peroxide block, polymer reagent (goat anti-rabbit horseradish peroxidase), mixed DAB (3,3′-diaminobenzidine) chromogen, and hematoxylin. The staining was performed at ambient temperature and working concentration Bond wash buffer (AR9590) or deionized water for washes. Briefly, the tissue section slides were baked at 60 °C for 30 min followed by Bond dewax (AR9222) and heat-induced epitope retrieval using the Bond Epitope Retrieval solution (AR9961) for 20 min. The endogenous peroxidase activity was blocked by incubation in peroxide block for 5 min. Protein blocking was performed by incubation with Leica PowerVision (PV6122) solution for 20 min, followed by incubation for 60 min with anti-CD4 (1:1,000, antibody diluent#8112) and polymer reagent for 15 min. The brown enzymatic color was developed after incubation with mixed DAB for 10 min. The tissue slides were counterstained with hematoxylin for 5 min, washed, dehydrated, and mounted with xylene-based mounting medium using the Leica Multistainer (ST5020) and Coverslipper (CV5030) program. Stained slides were fully digitized using the Hamamatsu NanoZoomer (S210) followed by visual quality control of the staining before digital image analysis (DIA). Cell-based immunopositivity counts were analyzed with the HALO (Indica Labs) algorithm within the defined region of interest (ROI) for each tissue sample. The tissue fold was excluded from the selected ROI, if applicable.

#### 2.9.2 Mouse plasma anti-KLH IgG ELISA

96-well plates (Corning) were coated with 50 μL of 2 μg/mL KLH peptide (Millipore Sigma 374807) in coating buffer (PBS + 10 mM MgCl_2_) overnight at 4 °C. Plates were washed with wash buffer (0.05% Tween in PBS) and then blocked with 2% bovine serum albumin in PBS blocking buffer for 1 h. Plasma samples were plated at a 1:30,000 dilution factor, and a standard curve was created with recombinant anti-KLH antibody (BioLegend). After washing, samples and standards were added for 2 h. After an additional wash step, detection antibody diluted 1:10,000 was added followed by incubation for 60 min. With a final wash step, TMB kit peroxide substrate was added to the plates for 30 min of incubation in the dark. Following incubation, the reaction was halted with commercial stop solution, and the plates were read on a Tecan Spark photometer at 450 nm absorbance. Antibody concentrations were calculated in μg/mL by interpolation from the standard curve in GraphPad Prism.

### 2.10 xGvHD mouse model

Female immunodeficient NSG mice (Jackson Labs) aged between 7 and 8 weeks were randomized according to their body weight using studyLog software and were irradiated at 200 rads. Initial treatment was administered on the same day. The comparator dose selection was based on dose titration to achieve pharmacologically active exposures, with 1 mg/kg selected as a representative dose for comparison. There were five treatment groups, eight animals per group: M5542–1 mg/kg; CTLA-4Ig 1 mg/kg; anti-OX40L 1 mg/kg; anti-OX40L 1 mg/kg + CTLA-4Ig 1 mg/kg; isotype control 2 mg/kg; and a control no GvHD group (n=4). Subsequent weekly treatments were administered intraperitoneally on the same day. Previously isolated and frozen human PBMCs (hPBMCs) were thawed and processed for engrafting in mice the following day, with mice receiving 10 × 10^6^ cells per animal via tail vein injection to induce the clinical and immunologic manifestations of GvHD. Following engraftment, treated mice were weighed and scored on activity level and fur texture three times per week. Blood samples were taken at approximately 8–10 days to confirm engraftment via flow cytometry. Mice were sacrificed when 20% of body weight was lost. Human IFNγ was measured in mouse plasma samples using an AlphaLISA kit (PerkinElmer Cat# AL217C) according to the manufacturer’s instructions. Plasma samples were analyzed in duplicate diluted 1:3 in 1× assay buffer supplied with AlphaLISA kit using a 20-µL assay format according to the manufacturer’s instructions in 96-well ½ area white plates. The AlphaLISA signal intensity was measured in an Envision plate reader (Perkin Elmer), and cytokine concentrations were calculated by interpolation from a standard curve using GraphPad Prism.

### 2.11 Statistical analysis

Means and standard error of the mean (SEM) were calculated for all data as appropriate. Data sets were normalized using min–max normalization in GraphPad Prism. Cytokine normalization for MDDC: T cell and MDDC: SLE PBMC MLR was calculated using MDDC: T only or MDDC: SLE PBMC only as the maximum and T cells alone or PBMCs alone as the minimum, respectively. The dose–response curves for IL-2, TNFα, and IL-6 in the MDDC: T cell assay were graphed in GraphPad Prism 8, and the IC_50_ value was determined using log(inhibitor) vs. response -- Variable slope (four parameters). Statistically significant differences in cytokine levels between groups in the MDDC: T cell MLR was determined using one-way analysis of variance (ANOVA) and Tukey’s test for multiple comparisons, while MDDC: SLE PBMC MLR assay was determined using two-way analysis of variance (ANOVA) and Tukey’s test for multiple comparisons. The frequency of Tregs in the iTreg induction assay was normalized to an isotype control, and statistically significant differences in frequency of FOXP3+CD25hi cells between samples were determined using one-way ANOVA. Statistically significant differences between samples in the KLH-DTH model were determined using one-way ANOVA with Tukey’s test for multiple comparisons. Ear thickness over time was analyzed using a mixed-effects model fitted by restricted maximum likelihood (REML) to account for repeated longitudinal measurements within the same animal and to accommodate missing values. Treatment group, time, and treatment × time interaction were included in the model.

Following DIA of the mouse ear tissue samples, the mean ± SEM percentage of CD4+ cells was calculated for each group and statistically significant differences determined using one-way ANOVA with Tukey’s test for multiple comparisons. Survival was analyzed using Kaplan–Meier survival curves and compared among groups using the log-rank (Mantel–Cox) test. The Gehan–Breslow–Wilcoxon test was performed as a complementary analysis to give greater weight to early events. Body-weight change over time was analyzed using a mixed-effects model (REML) to account for repeated longitudinal measurements within the same animal and to accommodate missing values. Treatment group, time, and treatment × time interaction were included as fixed effects. All statistical analyses were performed using GraphPad Prism. A two-sided *P* < 0.05 was considered statistically significant.

## 3 Results

### 3.1 M5542-modulated proinflammatory cytokine production and T-cell proliferation by disrupting the interaction of CD80, CD86, and OX40L on activated APCs to their cognate receptors on T cells in a mixed lymphocyte assay

To understand the potential biological and therapeutic significance of a dual CD28 and OX40 pathway blocker such as M5542 on reducing T-cell activation and effector function, we developed an MLR assay that models the inflammatory APC and T-cell interaction utilizing both CD28 and OX40 pathways for T-cell activation. In this inflammatory MLR, activated MDDCs, which expressed all three ligands (CD80, CD86, and OX40L), were cocultured with allogeneic T-cells, allowing the assessment of both CD28 and OX40 pathways contributing to T-cell and APC proinflammatory cytokine release and T-cell proliferation.

**Figure 1:**
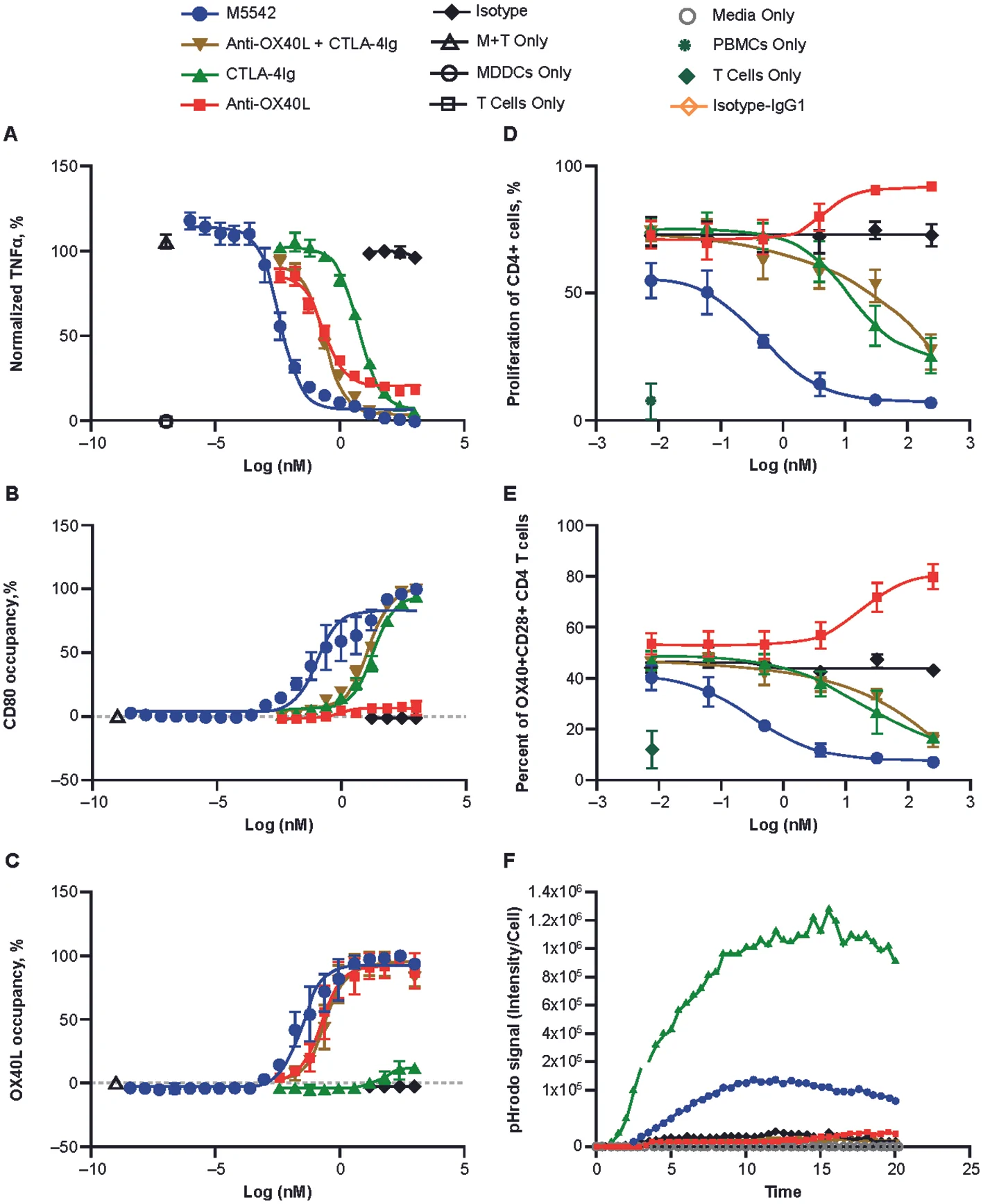
M5542 potently modulated proinflammatory cytokine production and T-cell proliferation by disrupting the interaction of CD80, CD86, and OX40L on activated APCs to their cognate receptors on T cells in a mixed lymphocyte assay. **(A)** Human MDDCs were cocultured with allogeneic T cells with various concentrations of the following test agents M5542, CTLA-4Ig, anti-OX40L, combination (anti-OX40L + CTLA-4Ig) or isotype control. Secretion of TNFα was assessed in the supernatant on day 4. Detection of free CD80 and OX40L on human MDDCs from the coculture was performed by flow cytometry and calculated as **(B)** CD80, and **(C)** OX40L receptor occupancy in the presence of increasing concentration of test agents. In a similar MLR of MDDCs and allogeneic PBMCs in the presence and absence of test agents, **(D)** proliferation of CD4+ T cells and **(E)** frequencies of OX40+CD28+CD4 T-cells were assessed. Error bars show average and SEM. The results were reproduced in 6 MLRs (n=6 T cell donors) for cytokine production and 2 MLRs (n=2 MDDCs) for receptor occupancy and 3 MLRs (n=3 T cell donors) for proliferation and OX40+CD28+CD4 frequencies. **(F)** pHrodo-labeled M5542, CTLA-4Ig, anti-OX40L, and negative control anti-CD20 (rituximab) or isotype control were tested at the 100-nM concentration to characterize their internalization to MDDCs over time as measured by live-cell imaging. The result shown was representative of two separate experiments of one donor MDDC. APC, antigen presenting cell; CTLA-4, cytotoxic T lymphocyte antigen-4; Ig, immunoglobulin; MDDC, monocyte-derived dendritic cell; MLR, mixed lymphocyte reaction; PBMC, peripheral blood mononuclear cell; SEM, standard error of the mean; TNFα, tumor necrosis factor alpha.

M5542 significantly and more potently reduced T-cell activation as evidenced by IL-2 at day 2 and proinflammatory cytokine production at day 4, with reductions in TNFα, IFNγ, and IL-6 levels compared with CTLA-4Ig or anti-OX40L alone (**Figure 1A**; **Supplementary Figure 2**; see Online Resource). M5542 was also more potent compared with the combination of the single agents (anti-OX40L + CTLA-4Ig) across all cytokines tested (**Figure 1A**; **Supplementary Figure 2**; see Online Resource). Furthermore, the reduction observed in all cytokines with M5542 at high concentrations was comparable with that observed in unstimulated controls. The 50% inhibitory concentration (IC_50_) values for inhibiting cytokine production with M5542 were significantly lower than with CTLA-4Ig alone, anti-OX40L alone, or the combination of the two (**Table 1**). Since CD80/CD86:CD28 signaling and OX40L:OX40 signaling both contribute to T-cell proliferation, we measured the effect of M5542 on reducing T-cell proliferation compared with single agents or the combination. Inflammatory MDDCs expressing the three ligands when cocultured with allogeneic PBMCs exhibited robust proliferation of CD4 and CD8 T cells as measured by dilution of CTV dye compared with unstimulated PBMCs alone. M5542 significantly decreased alloreactive CD4 and CD8 T-cell proliferation compared with single agents or the combination (**Figure 1D**; **Supplementary Figure 3A**; see Online Resource). Importantly, larger reduction in frequencies of costimulatory receptor-positive (OX40^+^CD28^+^) CD4 or CD8 were observed with M5542 than for CTLA-4Ig or anti-OX40L, suggesting a greater impact on activated T cells (**Figure 1E**; **Supplementary Figure 3B**; see Online Resource). CTLA-4Ig is known to suppress T-cell activation by blocking CD80/CD86:CD28-mediated costimulation, whereas anti-OX40L is known to block OX40L–OX40 axis-mediated T-cell expansion, survival, and memory formation. Accordingly, M5542 may act through complementary downstream pathways associated with both mechanisms (6).

**Table 1:** M5542 is an effective inhibitor of proinflammatory cytokines compared with anti-OX40L and CTLA-4Ig alone and in combination.

Cytokines	Average IC_50_ (nM) ± SEM			
	M5542	Combination	Anti-OX40L	CTLA-4Ig
IL-2	0.004 ± 0.0004***	1.1 ± 0.4***	NA	54 ± 25.9
TNFα	0.007 ± 0.002****	0.3 ± 0.05****	0.2 ± 0.04****	5.1 ± 0.4
IL-6	0.04 ± 0.01**	2.0 ± 0.9*	9.8 ± 9.5**	18.7 ± 4.9

One-way ANOVA was performed using Tukey’s multiple comparisons test for significance between groups. Significance compared with CTLA-4Ig, *P < 0.05; **P < 0.01; ***P < 0.001; ****P < 0.0001.

To understand the mechanism behind the improved efficacy of M5542 compared with single or combination pathway blockade, we evaluated changes in target expression of CD80, CD86, and OX40L on MDDCs by flow cytometry by each of the treatment conditions, as reflected by target occupancy. Compared with the isotype control, M5542 bound to CD80 and OX40L in a concentration-dependent manner, thereby occupying the targets and preventing activation of their cognate receptors CD28 and OX40, leading to reduced proinflammatory cytokine production (**Figures 1B, C**). M5542 had a 190-fold improved 50% receptor occupancy (RO_50_) with CD80 (0.1 nM) compared with CTLA-4Ig alone (19 nM), and a 10-fold improved receptor occupancy (RO_50_: 0.02 nM) with OX40L than with anti-OX40L alone (RO_50_: 0.2 nM). Interestingly, while blocking the two pathways in combination (CTLA-4Ig and anti-OX40L) generated RO_50_ values for CD80 (12.2 nM) and OX40L (0.3 nM) were similar to those achieved using the respective single agents, M5542 showed higher target coverage with 122-fold improved occupancy of CD80 and 15-fold improved occupancy of OX40L. CD86 occupancy could not be accurately determined due to a lack of competing staining antibody to M5542 and CTLA-4Ig.

To evaluate if the improved occupancy of M5542 for CD80 and OX40L over the single agents is due to the increased affinity of the M5542 fusion molecule for its targets, we measured the binding affinity of M5542 against CD80/CD86 and OX40L compared with the binding affinities achieved with CTLA-4Ig or anti-OX40L alone using SPR. M5542 exhibited binding affinities similar to those of the respective single agents in the steady-state affinity assay (**Table 2**).

**Table 2:** Binding affinities of M5542, anti-OX40L and CTLA-4Ig to targets by Biacore.

Agent	KD (nM)		
	Human OX40L	Human CD80	Human CD86
M5542	41	522	434
Anti-OX40L	50	NT	NT
CTLA-4Ig	NT	552	548
Isotype control	NB	NB	NB

NB, no binding; NT, not tested.

It is known that CTLA-4Ig is internalized upon binding to CD80/CD86, preventing further target engagement (34, 35). Since M5542 has both CTLA-4Ig and anti-OX40L components, we evaluated whether M5542 had a different target-mediated internalization profile to the single agents observed on MDDCs in the MLR assay. Test antibodies labeled with pHrodo dye can be tracked using live cell imaging on the MDDCs for increases in fluorescence when internalized into acidic organelles like endosomes and lysosomes. As expected, CTLA-4Ig was rapidly internalized, and internalization continued and plateaued over the 20 h of monitoring, while the isotype control, rituximab, and anti-OX40L remained low in pHrodo signal and were not internalized. M5542 showed limited internalization during the 20-h analysis (**Figure 1F**). The slower and reduced internalization of M5542 compared with CTLA-4Ig alone is likely due to the anti-OX40L component which did not internalize. The dual costimulation pathway blockade provided by M5542 reflected its unique biophysical properties, with both greater target occupancy and greater inhibition of T-cell proliferation and inflammation compared with each pathway blockade agent singly or in combination.

### 3.2 Comparable reductions in cytokine production with M5542 and single-pathway blockade agents in the SLE PBMC MLR

**Figure 2:**
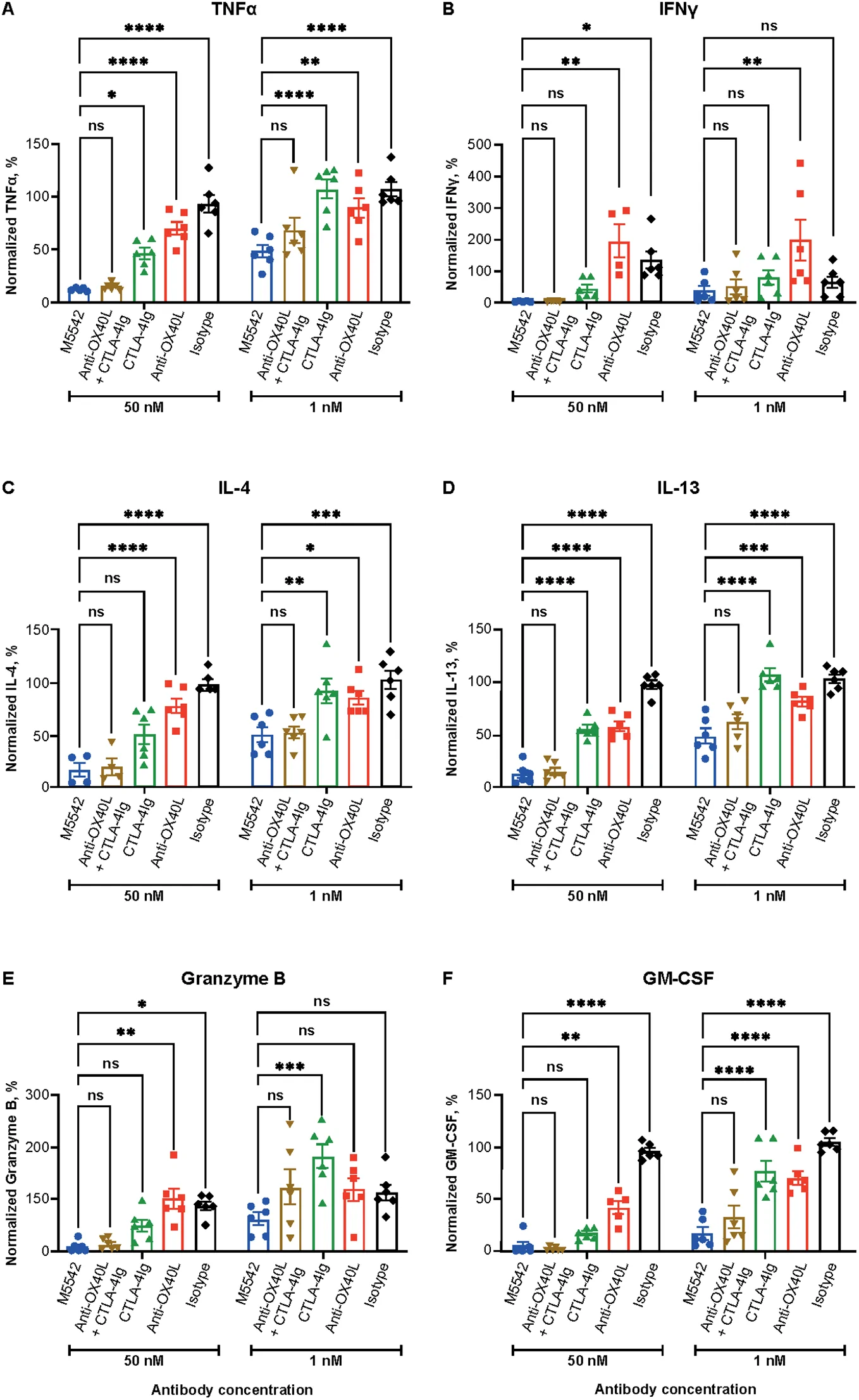
M5542 significantly reduced proinflammatory cytokine production in a human SLE PBMC *in vitro* model. MDDCs were cocultured with allogeneic SLE PBMCs in the presence of either 50 or 1 nM of test agent for 4 days. On day 4, supernatant was collected and assessed for **(A)** TNFα, **(B)** IFNγ, **(C)** IL-4, **(D)** IL-13, **(E)** Granzyme B, and **(F)** GM-CSF secretion. Results shown from three SLE PBMC MLRs performed in duplicate. Two-way ANOVA was performed using Tukey’s multiple comparisons test for significance between groups. ****P < 0.0001; ***P = 0.0002; **P = 0.0048; *P < 0.01; ns=not significant. Error bars show average and SEM. ANOVA, analysis of variance; GM-CSF, granulocyte-macrophage colony stimulating factor; IL, interleukin; IFNγ, interferon gamma; MDDC, monocyte derived dendritic cell; PBMC, peripheral blood mononuclear cell; SEM, standard error of the mean; SLE, systemic lupus erythematosus; TNFα, tumor necrosis factor alpha.

SLE is a heterogenous autoimmune disease, with subsets of patients having T-cell endotypes and activated T cells that drive disease pathology in the kidney, particularly in LN (36–40). To simulate an *in vitro* mechanistic model with patient immune cells, SLE PBMCs were used instead of using healthy donor T cells as responders in the allogeneic MLR assay. This *in vitro* mechanistic model enables assessment of the dual costimulation pathway blockade effects of M5542 and comparators to dampen additional cytokines. As expected, the effect of M5542 was also observed in this allogeneic MLR of MDDCs cocultured with SLE patient PBMCs. M5542 significantly reduced production of Teff cell cytokines such as TNFα, IFNγ, IL-4, IL-13, Granzyme B, and the MDDC cytokines such as GM-CSF at 50 nM compared to isotype control alone (**Figure 2**). Importantly, at the lower 1-nM concentration, significant differences in cytokine inhibition were also observed with M5542 compared with CTLA-4Ig or anti-OX40L alone for TNFα, IL-4, IL-13, and GM-CSF (**Figures 2A, C, D, F**). The effects of 1 nM M5542 on IFNγ were significantly lowered compared with anti-OX40L alone and were improved versus CTLA-4Ig alone on Granzyme B (**Figures 2B, E**). There were no significant differences between M5542 and the combination of each single agent (anti-OX40L+CTLA-4Ig) at the concentrations tested in this particular assay (**Figure 2**).

### 3.3 M5542 reverses OX40L-mediated suppression of induced T-regulatory-cell generation and reduces T-effector-cell proliferation in T-regulatory-cell-containing cocultures

**Figure 3:**
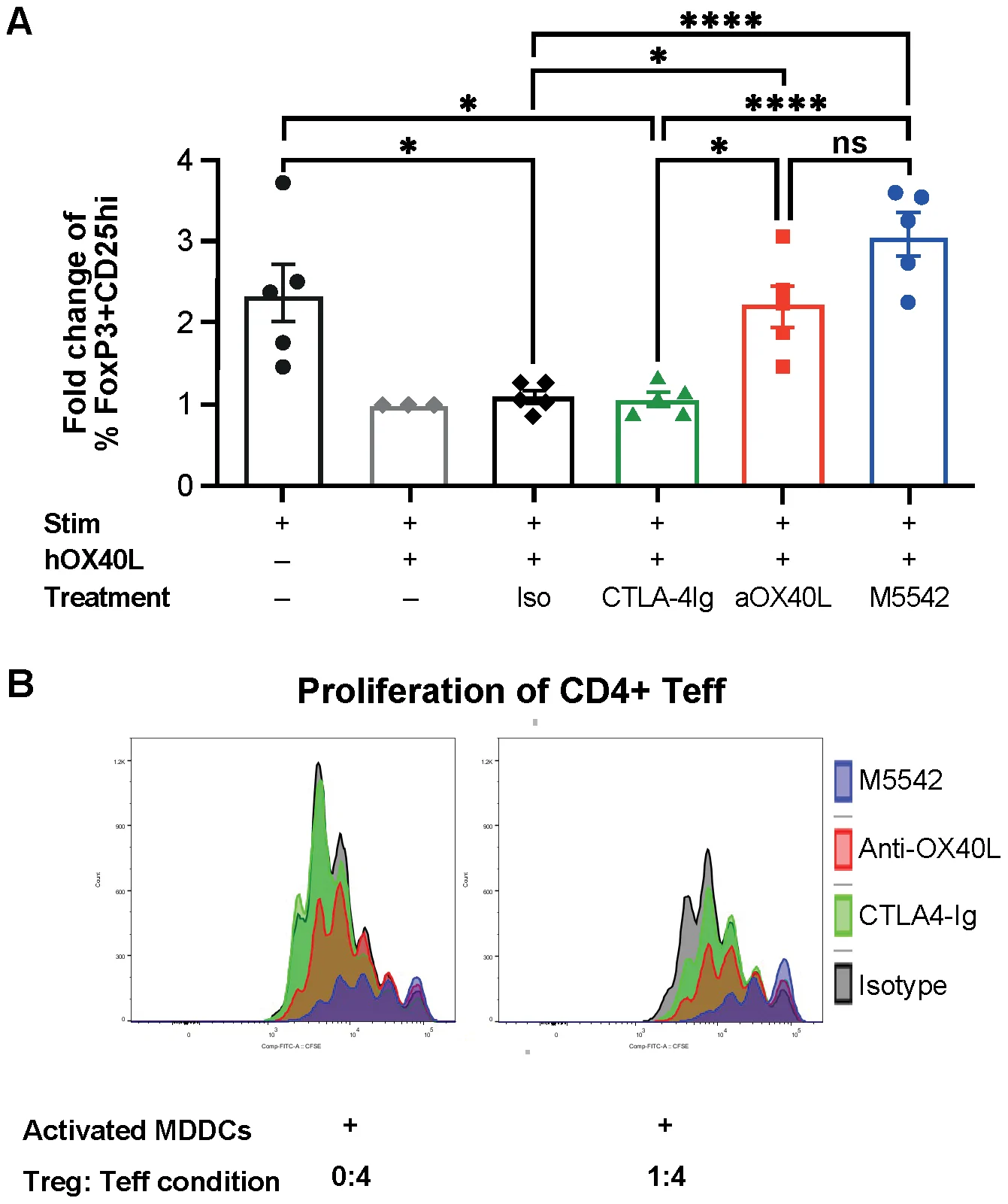
M5542 reverses OX40L-mediated suppression of induced T-regulatory-cell generation and reduces T-effector-cell proliferation in T-regulatory-cell-containing cocultures. **(A)** Human naïve CD4 T cells were cultured with Treg induction cocktail in the presence or absence of soluble human OX40L and test antibodies for 5 days. %FOXP3+CD25+ of CD4+CD25+CD127lo Tregs were quantified by flow cytometry and reported as fold change compared with isotype control. Ordinary one-way ANOVA was performed using Dunnett’s multiple comparisons test for significance between groups. *P < 0.01; ****P < 0.0001; ns = not significant. Error bars show average and SEM. (n=5). **(B)** Human CD4 T cells were sorted into CD127^lo^CD25^hi^ (Treg), while remaining CD25+CD127+ non-Tregs (Teff) cells were labeled with a proliferation tracker (CFSE). Human MDDCs cocultured with allogeneic Teff in the absence (0:4 ratio) or presence of Tregs and Teff in a 1:4 ratio was treated with test agents for 4 days. The effects of test agents on Teff proliferation in either condition were assessed using flow cytometry. Results shown are representative of two MLRs. ANOVA, analysis of variance; MDDC, monocyte derived dendritic cell; PBMC, peripheral blood mononuclear cell; SEM, standard error of the mean; Teff, T-effector cell; Treg, T-regulatory cell.

Serum soluble OX40L levels and expression of OX40L on APCs are known to be significantly elevated in patients with active SLE and LN compared with healthy controls (26, 41). OX40L-mediated OX40 signaling has been shown to block Treg generation due to direct downregulation of FOXP3 expression, implicating the pathogenic role of OX40L in Treg dysfunction observed in SLE (9, 24, 25, 42). In an iTreg cell differentiation assay, as reported in the literature, the addition of recombinant soluble OX40L significantly reduced iTreg cell numbers characterized as CD25+CD127dimFOXP3^+^ compared with induction media alone (**Figure 3A**). Since this assay is driven by soluble OX40L, as expected, both M5542 and anti-OX40L treatment blocked the effect of soluble OX40L and reversed the detrimental effect on iTreg generation, while isotype control or CTLA-4Ig alone had no effect (**Figure 3A**).

Other than its negative impact on Treg generation, OX40L has also been implicated in negatively affecting Treg suppressive function, through downregulation of FOXP3 expression in Tregs in SLE (9, 25). To evaluate antigen-specific Treg-suppressive function on Teff cells, we developed a three-way coculture of activated MDDCs with allogeneic Teff and Tregs where the T cells were sorted from the same donor. Treg suppression assays were performed using Tregs and Teff cells derived from healthy donors. Here, the MDDCs provided the CD80/CD86:CD28 and OX40L:OX40 pathway signaling to induce the activity of allogeneic Teff and Tregs, and the net effect of Tregs and the influence of M5542 treatment on Teff proliferation were observed. To test whether M5542 could effectively target Teff proliferation and allow Treg suppressive activity compared with the single agents, we evaluated how each treatment affected Teff proliferation in the absence or presence of Tregs. As expected, in the MLR without Tregs, M5542 was more effective in reducing Teff proliferation than CTLA-4Ig or anti-OX40L alone. The addition of Tregs in the MLR showed that Tregs were able to suppress Teff proliferation as seen with isotype control. Moreover, the presence of Tregs and treatment with CTLA-4Ig or anti-OX40L suppressed Teff proliferation more than Tregs alone. In comparison, M5542 provided greater suppression of Teff cell proliferation in the presence of Treg cells than the single agents (**Figure 3B**). This result suggested that there was no evidence that M5542 inhibited Treg function, rather that M5542 activity and Treg functional activity can contribute to suppressing Teff cell proliferation in the context of this three-way MLR. These observations indicate that M5542 potentially prevents OX40L-mediated Treg dysfunction and that M5542 and Tregs may work jointly to further decrease Teff proliferation, particularly in an antigen-specific manner involving APCs such as MDDCs.

### 3.4 M5542 effect on antigen-specific T-cell inflammation and T-dependent antibody response in a KLH-DTH model

**Figure 4:**
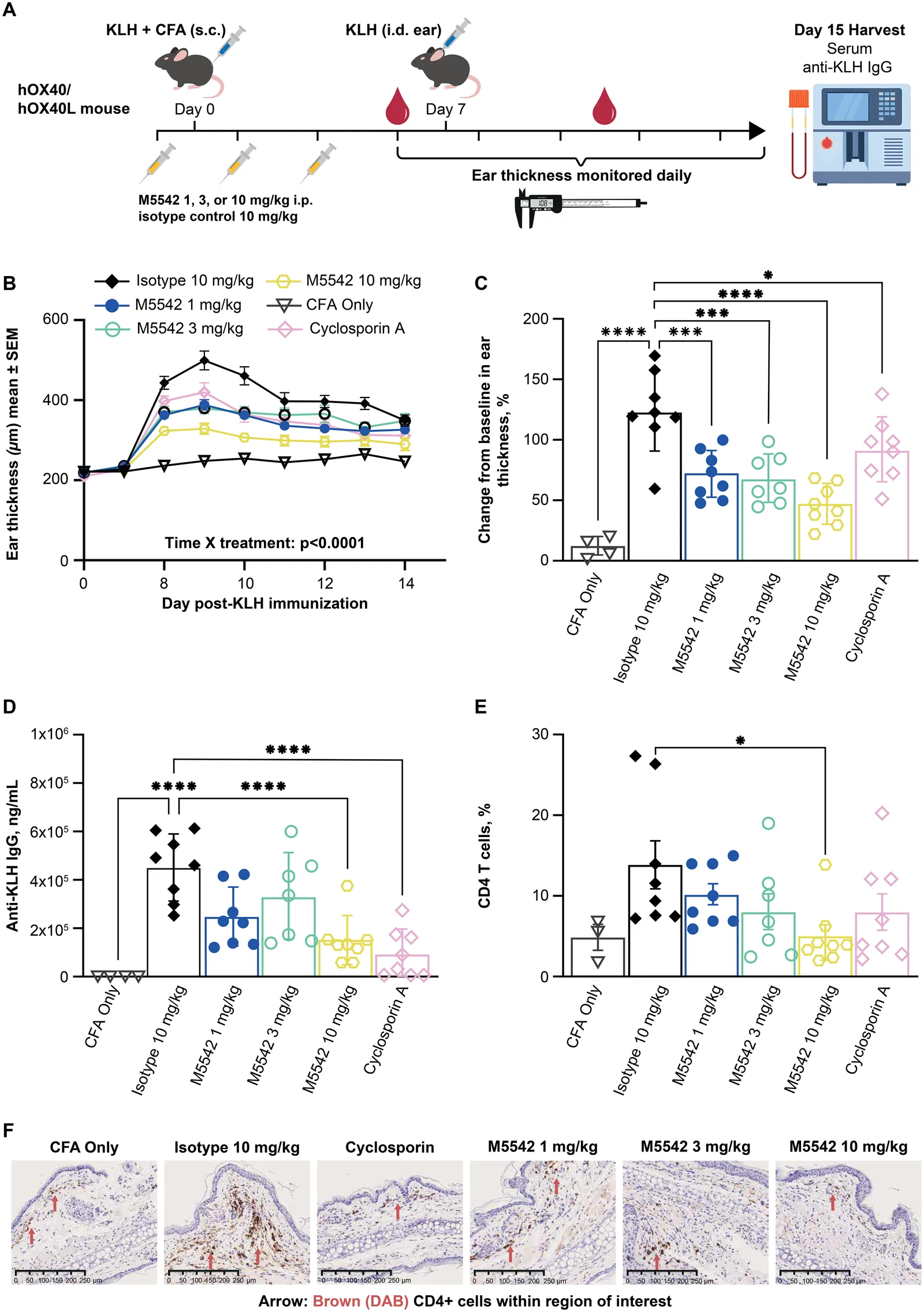
M5542 inhibits ear swelling and anti-KLH IgG induction in a KLH-DTH model. **(A)** Model schematic showing human OX40/OX40L transgenic mice administered on day −1 prior to subcutaneous KLH/CFA immunization at day 0 and KLH peptide ear injection on day 7. Mice were dosed i.p. three times per week through day 13 with isotype 10 mg/kg, M5542–1 mg/kg, 3 mg/kg, and 10 mg/kg, and cyclosporin A 60 mg/kg (oral daily). **(B)** Ear swellings were measured at baseline and tracked daily post ear injection. **(C)** Peak ear swelling at day 9 was used to compare all groups to baseline ear thickness. **(D)** Plasma anti-KLH IgG was measured on day 15. **(E)** Percent and **(F)** representative images of CD4+ cell staining on fixed ear tissue samples from day 15 post-immunization. Ear thickness over time following KLH immunization in the indicated treatment groups. Data are presented as mean +/- SEM. Longitudinal ear-thickness measurements were analyzed using a mixed-effects model to account for repeated measurements within animals. Significant effects of time (*P* < 0.0001), treatment group (*P* < 0.0001), and time × treatment interaction (*P* < 0.0001) were observed. Ear thickness at day 9 and plasma anti-KLH IgG are shown as the average with SEM. Statistics were calculated by one-way ANOVA with Tukey’s multiple comparisons. Comparison to isotype shown as *P < 0.05; ***P < 0.001; ****P < 0.0001. All groups have n=8 except CFA control n=4. ANOVA, analysis of variance; CFA, complete Freund’s Adjuvant; DTH, delayed-type hypersensitivity; IgG, immunoglobulin G; KLH, keyhole limpet hemocyanin; SEM, standard error of the mean.

The KLH peptide-induced DTH mouse model exhibits a robust T-cell dependent antibody response (TDAR) and T-cell mediated inflammation of the ear, providing a useful model to study immunomodulatory compounds *in vivo* (43). Since the anti-OX40L arm of M5542 is not cross-reactive to mouse OX40L, we leveraged transgenic mice expressing human OX40 and human OX40L to set up the KLH-DTH model (**Figure 4A**). Treatment with M5542 in this model reduced the KLH-induced ear swelling in a dose-dependent manner compared with the isotype control, with M5542–10 mg/kg achieving greater reduction than cyclosporin A in this model (**Figures 4B, C**). Mixed-effects analysis of longitudinal ear-thickness measurements revealed significant effects of time (*P* < 0.0001), treatment group (*P* < 0.0001), and time × treatment interaction (*P* < 0.0001), indicating that ear-thickness trajectories differed significantly among groups over time. In addition, the anti-KLH IgG TDAR response was inhibited significantly by M5542 at 10 mg/kg, as well as by the cyclosporin A-positive control, compared with isotype levels (**Figure 4D**). Moreover, M5542 treatment led to a reduction in CD4 T-cell infiltration into the ear by histology at day 15 after immunization compared with controls (**Figures 4E, F**). The results showed that M5542 was efficacious in inhibiting T-cell-mediated inflammation in the ear and anti-KLH IgG TDAR responses in the human OX40/OX40L Tg KLH-DTH model.

### 3.5 M5542 effect on inhibiting xenogeneic T-cell responses in a xGvHD mouse model

**Figure 5:**
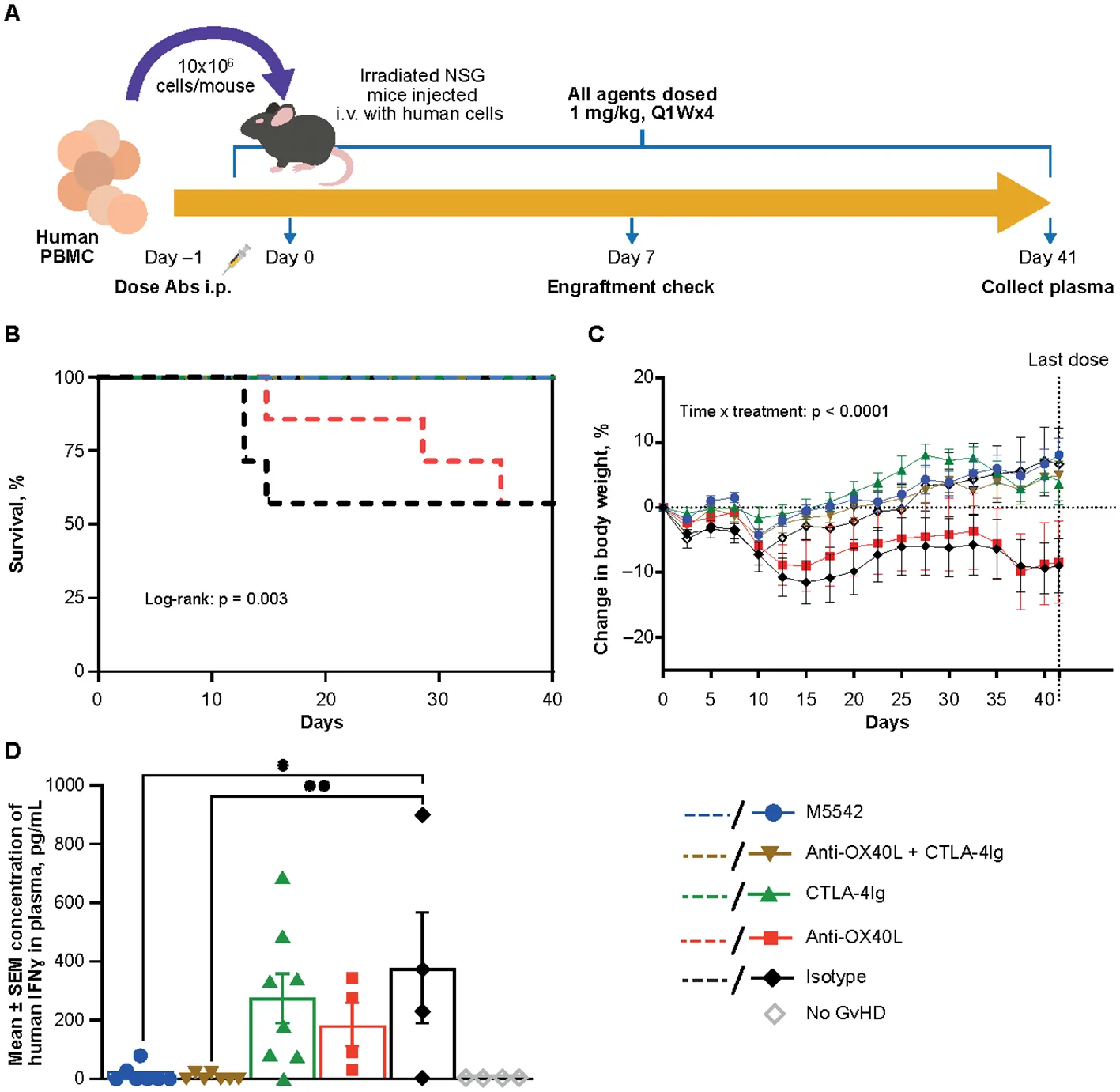
M5542 demonstrated improved efficacy in an xGvHD disease mouse model. **(A)** Irradiated NSG mice were dosed with M5542, CTLA-4Ig, anti-OX40L at 1 mg/kg, or combination of anti-OX40L 1 mg/kg + CTLA-4Ig 1 mg/kg, once per week i.p. compared with isotype control (2 mg/kg) starting 1 day prior to human PBMC engraftment. **(B)** Kaplan–Meier survival analysis of the indicated treatment groups. Survival was monitored over time and plotted as percent survival. Survival distributions differed significantly among groups by log-rank (Mantel–Cox) analysis (*P* = 0.0030). Similar results were obtained using the Gehan–Breslow–Wilcoxon test (*P* = 0.0031). Percent survival was recorded based on mice succumbing to disease upon 20% weight loss threshold or death. **(C)** Weight loss was tracked 3 days per week. Body-weight changes over time in the indicated treatment groups. Data are presented as mean +/− SEM. Longitudinal body-weight measurements were analyzed using a mixed-effects model to account for repeated measurements within the same animal. Significant effects of time (*P* < 0.0001), treatment group (*P* = 0.0146), and time × treatment interaction (*P* < 0.0001) were observed. **(D)** Plasma IFNγ cytokine levels were measured on day 41. Data are shown as mean with SEM. Statistics were calculated by one-way ANOVA with Tukey’s multiple comparisons. Comparison to isotype shown as *P < 0.05; ***P < 0.001; ****P < 0.0001. For B-C: All groups have n=8, except no GvHD control n=4. For D: M5542 (n=7), CTLA-4Ig (n=8), anti-OX40L (n=4), anti-OX40L+ CTLA-4Ig (n=7), isotype (n=4), and no GvHD (n=4) because some animals did not survive until day 41 for this measurement ANOVA, analysis of variance; CTLA-4, cytotoxic T lymphocyte antigen-4; IFNγ, interferon gamma; Ig, immunoglobulin; i.p., intraperitoneal; i.v., intravenous; PBMC, peripheral blood mononuclear cell; SEM, standard error of the mean; xGvHD, xenogeneic graft-versus-host disease.

To evaluate the efficacy of M5542 in a disease model, a xGvHD model was developed in humanized NSG mice (NOD-*scid* IL2Rgamma^null^) reconstituted with hPBMCs (**Figure 5A**). Upon reconstitution in mice, human immune cells first populate secondary lymphoid organs, where they undergo xeno-antigen priming, and then home toward and infiltrate non-lymphoid GvHD-target organs. Upon infiltration, these human cells induce the immunologic and clinical manifestations of GvHD, including widespread organ damage which can be manifested by weight loss, increased levels of cytokines in plasma, and ultimately reduced survival.

Treatment with M5542 in the xGvHD mouse model improved survival, with 100% survival observed after 41 days. Kaplan–Meier analysis demonstrated a significant difference in survival among treatment groups, as assessed by the log-rank (Mantel–Cox) test (*P* = 0.0030), with concordant results obtained using the Gehan–Breslow–Wilcoxon test (*P* = 0.0031). Survival with M5542 was improved compared with anti-OX40L alone and was comparable to that achieved with CTLA-4Ig alone or in combination with anti-OX40L (**Figure 5B**). A similar result was seen for body weight, with prevention of weight loss observed in the M5542, CTLA-4Ig, and anti-OX40L+CTLA-4Ig groups, while mice receiving the isotype control or anti-OX40L alone lost approximately 10% of body weight (**Figure 5C**). Mixed-effects analysis of longitudinal body-weight measurements revealed significant effects of time (*P* < 0.0001), treatment group (*P* = 0.0146), and time × treatment interaction (*P* < 0.0001), indicating that body-weight trajectories differed among treatment groups over time. M5542 also significantly inhibited the production of plasma IFNγ compared with the isotype control treatment; the effect was greater than that observed in either the CTLA-4Ig or anti-OX40L monotherapy groups (which did not achieve significance), and similar to that observed in mice receiving the combination treatment of anti-OX40L and CTLA-4Ig (**Figure 5D**). In conclusion, M5542 was effective in improving survival and body weight and inhibiting plasma IFNγ levels in mice compared with CTLA-4Ig alone or anti-OX40L alone in mice with xGvHD. M5542 as a bifunctional molecule (**Supplementary Figure 1**) achieved the same effect as the combination of CTLA-4Ig and anti-OX40L as single agents in a T-cell-mediated disease model.

## 4 Discussion

In this study, we demonstrated that blockade of the costimulatory molecules CD80/86 and OX40L by the bifunctional fusion molecule M5542 effectively inhibited T-cell activation, proliferation, and proinflammatory cytokine production. Moreover, M5542 neutralized OX40L-driven Treg dysregulation and suppressed Teff in conjunction with Tregs *in vitro*. Additionally, in an *in vivo* mouse model of GvHD, M5542 improved survival, prevented weight loss, and effectively inhibited production of the proinflammatory cytokine IFNγ, while in a KLH peptide-induced DTH mouse model, M5542 can significantly inhibit T-cell-mediated inflammation in the ear at lower doses but a higher dose is needed to inhibit T-dependent antibody responses. We also demonstrated that the effects achieved with M5542 are generally greater than those observed with either CTLA-4Ig or anti-OX40L alone, and at least comparable to both agents in combination.

In MLR assays, M5542 effectively blocked both CD28 and OX40 pathways and successfully inhibited T-cell activation and proinflammatory cytokine production, specifically IL-2, TNFα, and IFNγ. These cytokines are produced by Th1 cells, and enhanced Th1 responses have been implicated in autoimmunity, contributing to immunopathology in several animal models including experimental autoimmune encephalomyelitis (44), RA (45), and GvHD (46). Blockade of OX40L-OX40 signaling has been shown to reduce disease in these models (44, 46) and has demonstrated efficacy in a successful Phase 2 study in patients with atopic dermatitis (31, 32), arising from the potential of OX40L-OX40 blockade to reduce both memory T-cell survival and antigen-specific T-cell differentiation. Similarly, the results observed here are consistent with those observed during costimulation blockade with CTLA-4Ig, which inhibited T-cell activation (15) and reduced T-cell proliferation and the production of proinflammatory cytokines (47). This activity has led to the approval for CTLA-4Ig in several autoimmune diseases, including RA (13). In addition, M5542 decreased the frequencies of OX40^+^CD28^+^ CD4/CD8 and reduced CD4/CD8 T-cell proliferation more potently than either CTLA-4Ig or anti-OX40L alone. This finding may suggest a potential benefit in chronic inflammatory diseases where *in vivo* expansion CD28^−^CD4^+^ T cells has been observed in patients with autoimmune disease, including RA (48), SLE (49), and multiple sclerosis (50). These cells are thought to require other costimulatory pathways, such as OX40/OX40L (18), which can be targeted by M5542. Unfortunately, these CD28null T cells are difficult to model *in vitro* as majority of cells in the MLR were CD28^+^OX40^+^ not OX40^+^CD28^−^ T cells due to culture conditions.

Either CTLA-4Ig or anti-OX40L alone inhibited cytokine production in MLR assays, but neither could maintain this inhibition at lower concentrations, suggesting that dual blockade via M5542 may provide more effective inhibition. Furthermore, M5542 reduced cytokine production to baseline levels in unstimulated MDDCs only or T cells only, as well as demonstrating greater potency in reducing cytokine production compared with CTLA-4Ig or anti-OX40L alone, and interestingly better than the combination of CTLA-4Ig and anti-OX40L in some assays. This added potency could be driven by increased receptor occupancy of CD80 and OX40L observed in the MLR assays. Although M5542 bound to soluble targets CD80/CD86 and OX40L with similar affinities to those of CTLA-4Ig and anti-OX40L respectively, its occupancy of CD80 was greater than that of CTLA-4Ig alone, with an approximate 190-fold increase and a 10-fold greater occupancy of OX40L than anti-OX40L alone. Interestingly, greater occupancy of CD80 and OX40L was observed with M5542 over two single-pathway blockade agents given in combination, suggesting that the bispecific binding of the antibody is imparting an avidity effect on the binding of M5542 to its targets. Moreover, M5542 showed reduced target-mediated internalization into MDDCs compared with CTLA-4Ig, which also contributes to the improved target occupancy of CD80 and OX40L and the improved cytokine inhibition observed. Tregs constitutively express CTLA-4 which can serve to rapidly inhibit CD80/CD86 costimulation effects on Teffs (51). In the autoimmune disease context where Tregs are dysregulated or dysfunctional, leading to excessive activation of the CD28 pathway on Teffs, M5542 may be more advantageous or efficacious than CTLA-4Ig to inhibit the target ligands.

We also performed MLR assays using blood samples derived from patients with SLE. Both the OX40L-OX40 and CD80/CD86-CD28 costimulation pathways are critical to T-cell activation, proliferation, and differentiation and are known to be upregulated in patients with SLE (52, 53). The ligands in these two pathways therefore represent potential targets for intervention in treating subsets of SLE with T-cell endotype or LN where activated T cells are known to contribute to kidney pathology (36–40, 53). We demonstrated that M5542 effectively blocked both CD28 and OX40 pathways and successfully blocked T-cell activation and proinflammatory cytokine production at both high and low concentrations compared to isotype control. While CTLA-4Ig alone or anti-OX40L alone can reduce MLR-mediated TNFα, IL-4, IL-13, and GMCSF cytokine production at high concentration but not at low concentration, M5542 can reduce these cytokine levels at either high or low concentration. The cytokines evaluated in this study are representative of Th1, Th2, cytotoxic Th1, or CD8 T cells often described as pathogenic in various autoimmune diseases. These data suggest that M5542, with the additional mechanism of OX40L-OX40 pathway blockade, may provide deeper and broader impact and potentially better efficacy in reducing differentiation and cytokine production in various T-cell subsets compared to abatacept, which failed to meet its clinical endpoint in SLE and LN.

In an autoimmune setting, Tregs that often control chronically activated Teff cells are reduced or become dysfunctional. Our results suggest that M5542 has the potential to correct this imbalance. The OX40L-OX40 pathway signaling enhances Teff function while suppressing development of Tregs due to direct suppression of FOXP3 gene expression (24, 25). The impact of OX40L-OX40 signaling on reducing FOXP3+ Tregs was modeled in an *in vitro* Treg induction assay. Treg generation was suppressed by soluble OX40L whereas its neutralization with M5542 or anti-OX40L, but not with CTLA-4Ig or isotype control, showed that Treg generation can be modulated to levels where soluble OX40L was absent in this assay. Additionally, a three-way MLR was employed to assess the effects of M5542 on Treg and Teff cells, demonstrating improved inhibition of Teff cell proliferation *in vitro*.

Given that OX40L–OX40 signaling has been reported to impair Treg function via downregulation of FOXP3 and attenuating suppressive function, we interpret that blocking this pathway by M5542 may contribute to restoring Treg suppressive capacity (25). This is consistent with our findings, as M5542 more effectively inhibited Teff proliferation in Treg-containing assays compared with CTLA-4Ig or anti-OX40L. However, we acknowledge that the reduced effector proliferation may also reflect direct inhibition of effector activation rather than the direct evidence of enhanced restoration of Treg suppression.

To confirm the effectiveness of M5542, we conducted an *in vivo* assessment of the efficacy of M5542, in two T-cell-mediated disease animal models. Since M5542 does not cross-react with mouse OX40L, human OX40/OX40L transgenic mice and humanized mice were utilized. It has been reported that CTLA-4Ig (functionally similar to the CTLA-4 component of M5542) cross-reacts with mouse CD80 and CD86 (54). The first model was a KLH-DTH model in human OX40/OX40L mice, which simulated skin inflammation mediated by KLH-specific T-cell responses and systemic *de novo* T-dependent anti-KLH antibody responses. M5542 reduced ear inflammation/swelling in a dose-dependent manner without affecting *de novo* KLH antibody responses at low doses (1 and 3 mg/kg). As expected, higher doses (10 mg/kg) of M5542 suppressed both ear swelling and *de novo* generation of anti-KLH IgG, which requires costimulation and T-cell help.

The second model was a humanized xGvHD model (55). In this model, human donor cells infiltrate non-lymphoid GvHD-target organs, inducing the immunologic and clinical manifestations of GvHD, including widespread organ damage, which can be manifested by weight loss, increased levels of cytokines in plasma, and reduced survival. Weekly administration of M5542 prevented weight loss and maintained 100% survival throughout the study. IFNγ levels were significantly suppressed when compared with anti-OX40L and CTLA-4Ig alone, indicating that in this *in vivo* model, M5542 was effective at preventing the immune-driven pathology seen in GvHD. In both models, M5542 reduced disease biomarkers and cytokines and showed greater efficacy than either single-agent blockade alone in ameliorating disease development.

### 4.1 Limitations of the study

This study has some limitations that should be considered when interpreting the results. The non-depleting and non-cytotoxic feature of M5542 is inferred from its engineered Fc design. For target occupancy analysis of MDDC in the MLR, CD86 target occupancy could not be quantified owing to the absence of a suitable competing staining antibody. In the Treg induction assay, we used anti-CD28–mediated costimulation, which bypasses CD80/CD86 engagement, limiting this assay to OX40L-OX40 pathway readouts. Treg suppression assays were performed using healthy donor-derived cells rather than SLE patient-derived populations, and healthy donor MDDCs were used as APCs alongside SLE PBMCs due to limited patient sample availability. Finally, as M5542 binds selectively to human OX40L without cross-reactivity to murine OX40L, evaluation was restricted to a humanized OX40/OX40L transgenic mice and xGvHD model that has limited myeloid and B-cell activity. Studies in more advanced human immune reconstitution systems, such as CD34^+^ HSC-engrafted models (56), will be required for comprehensive dual-pathway assessment. Additionally, broader evaluations of safety, pharmacokinetics, and exposure–response relationships were beyond the scope of the current *in vitro* and *in vivo* studies and warrant further investigation.

**Figure 6:**
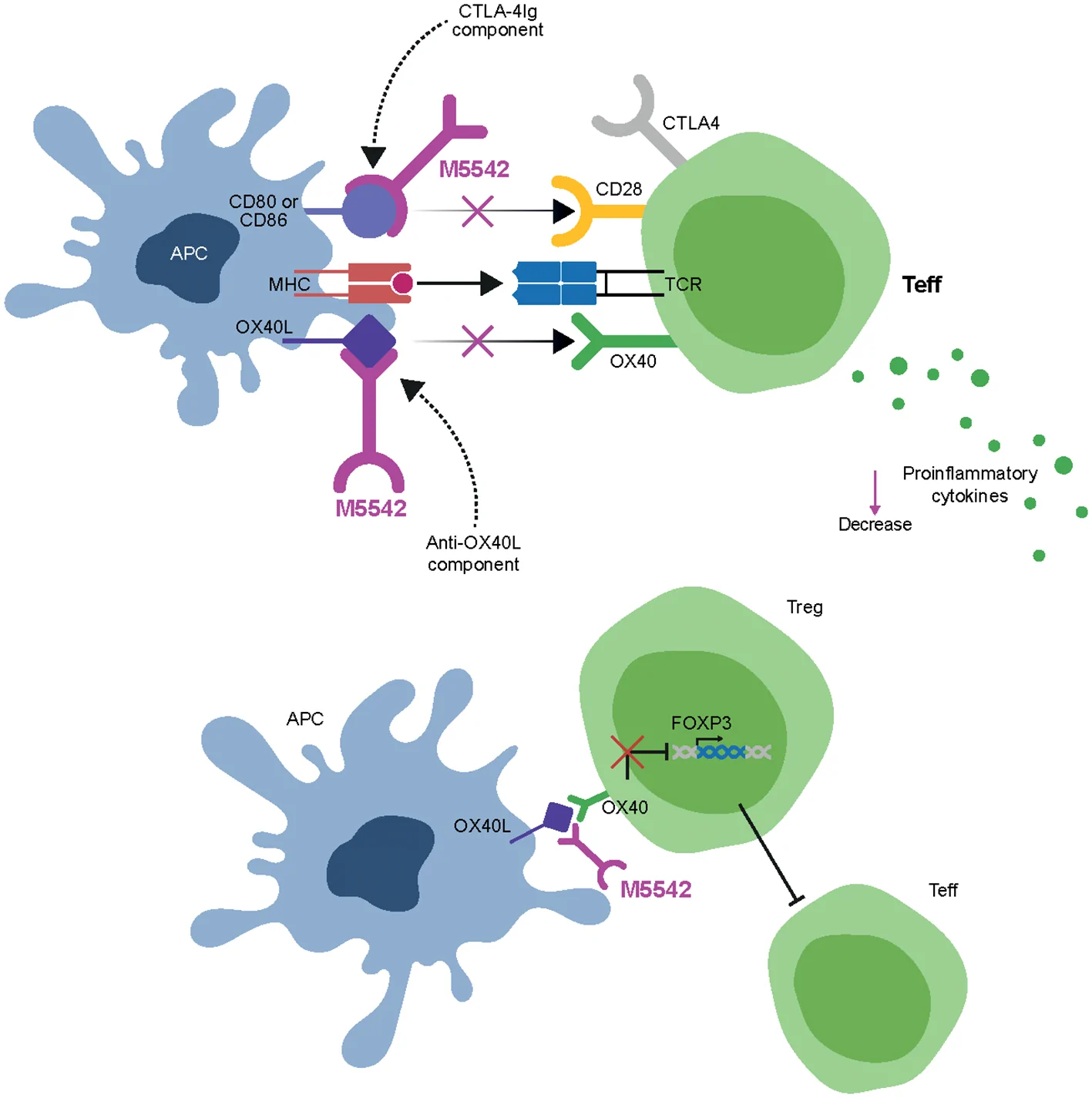
Proposed schematic of M5542 activity. M5542 is a bifunctional fusion protein which binds CD80/CD86 and OX40L on the surface of APCs, preventing their respective interaction with cognate CD28 and OX40 receptors expressed on T cells. This dual pathway blockade by M5542 potently reduces proinflammatory cytokines from Teffs (Th1, Th2) and APCs (IL-6, GM-CSF) but also T-cell proliferation. Since OX40L:OX40 signaling directly reduces generation of FOXP3+ Tregs, M5542 neutralizes OX40L to restore Treg generation and works jointly with Tregs to inhibit Teff proliferation. APC, antigen-presenting cell; CTLA-4, cytotoxic T lymphocyte antigen-4; FOXP3, forkhead box P3; GM-CSF, granulocyte-macrophage colony stimulating factor; Ig, immunoglobulin; IL-6, interleukin-6; Teff, T-effector cell; Th, T helper cell; Treg, T-regulatory cell.

In conclusion, the bifunctional fusion protein, M5542, effectively binds CD80/CD86 and OX40L on APCs preventing APC:T-cell crosstalk, thereby reducing proinflammatory cytokine production *in vitro* better than CTLA-4Ig or anti-OX40L alone. Additionally, M5542 demonstrated efficacy comparable to or greater than the combination of CTLA-4Ig and anti-OX40L in some assays. Moreover, M5542 neutralized the OX40L-associated Treg dysregulation, potentially impacting Treg function and concurrently reduced Teff proliferation in coculture (**Figure 6**). Importantly, M5542 was efficacious *in vivo* in an xGvHD model, improving survival, preventing weight loss, and inhibiting production of the proinflammatory cytokine IFNγ. These results provide proof of concept of the dual-blockade approach and showcased the immunomodulatory activity of M5542. Cumulatively, the results of this preclinical study warrant further investigation of M5542 as a potential therapeutic agent for the management of certain T-cell-mediated autoimmune diseases.

## Data Availability

The data supporting this study are not publicly available for legal and commercial reasons. Requests to access the datasets should be directed to the corresponding author.
