# Supplementary material for: M5542, a bifunctional fusion protein targeting CD80, CD86, and OX40L that modulates excessive T-cell activity: a preclinical proof-of-concept study
Source: Front Immunol. 2026 Aug 26;17:1809074. doi: 10.3389/fimmu.2026.1809074 (PMC13553540; doi:10.3389/fimmu.2026.1809074)
Supplement: Supplementary file 1 [file Table1.docx]

Supplementary Material

# 1 Supplementary Figures

## 1.1 Supplementary Figure 1

**Supplementary Figure 1.** **Schematic illustration of M5542 composition.** The ECD of CTLA-4 (filled purple rectangle) was fused to the N-terminus of a variant IgG1 Fc domain containing mutations that reduce Fc effector function (open rectangle), disulfide bonds are indicated in yellow, and anti-OX40L Fab domains (VH and VL, blue; CH1 and CL, red) were fused to the C-terminus of the variant IgG1 Fc domain.

CTLA-4, cytotoxic T lymphocyte antigen-4; Fab, antigen binding fragment; Fc, fragment crystallizable; ECD, extracellular domain; IgG, immunoglobulin.

## 1.2 Supplementary Figure 2

**A**

**B**

**C**

**Supplementary Figure 2.** M5542 exhibits greater potency on modulating proinflammatory cytokines IL-2 (A), IFNɣ (B), IL-6 (C) than single agents or combination.

CTLA-4, cytotoxic T lymphocyte antigen-4; IL, interleukin; IFNɣ, interferon gamma.

## 1.3 Supplementary Figure 3

**Supplementary Figure 3.** M5542 exhibits greater potency on inhibiting CD8 T-cell proliferation (A) and correspondingly reduced OX40+CD28+ CD8 T-cells (B).

CTLA-4, cytotoxic T lymphocyte antigen-4; Ig, immunoglobulin.

## 1.4 Supplementary Figure 4

**Supplementary Figure 4.** Human PBMC engraftment in the xGvHD model at Day 7

CTLA-4, cytotoxic T lymphocyte antigen-4; Ig, immunoglobulin; GvHD, graft-vs-host disease
